# Reductive Amination Reaction for the Functionalization of Cellulose Nanocrystals

**DOI:** 10.3390/molecules26165032

**Published:** 2021-08-19

**Authors:** Omar Hassan Omar, Rosa Giannelli, Erica Colaprico, Laura Capodieci, Francesco Babudri, Alessandra Operamolla

**Affiliations:** 1 Istituto di Chimica dei Composti Organo Metallici (ICCOM), Section of BARI, Consiglio Nazionale delle Ricerche (CNR), Via Edoardo Orabona 4, I-70126 Bari, Italy; hassan@ba.iccom.cnr.it; 2 Dipartimento di Chimica, Università degli Studi di Bari Aldo Moro, Via Edoardo Orabona 4, I-70126 Bari, Italy; rossella-giannelli@libero.it (R.G.); ericacolaprico@gmail.com (E.C.); francesco.babudri@uniba.it (F.B.); 3 Laboratory for Functional Materials and Technologies for Sustainable Applications (SSPT-PROMAS-MATAS), ENEA-Italian National Agency for New Technologies, Energy and Sustainable Economic Development, S.S. 7 “Appia” km 706, I-72100 Brindisi, Italy; laura.capodieci@enea.it; 4 Dipartimento di Chimica e Chimica Industriale, Università di Pisa, Via Giuseppe Moruzzi 13, I-56124 Pisa, Italy

**Keywords:** cellulose nanocrystals, nanocellulose, pyrene, organic light-emitting material, reductive amination, nanocellulose functionalization, nanocellulose fluorescence

## Abstract

Cellulose nanocrystals (CNCs) represent intriguing biopolymeric nanocrystalline materials, that are biocompatible, sustainable and renewable, can be chemically functionalized and are endowed with exceptional mechanical properties. Recently, studies have been performed to prepare CNCs with extraordinary photophysical properties, also by means of their functionalization with organic light-emitting fluorophores. In this paper, we used the reductive amination reaction to chemically bind 4-(1-pyrenyl)butanamine selectively to the reducing termini of sulfated or neutral CNCs (S_CNC and N_CNC) obtained from sulfuric acid or hydrochloric acid hydrolysis. The functionalization reaction is simple and straightforward, and it induces the appearance of the typical pyrene emission profile in the functionalized materials. After a characterization of the new materials performed by ATR-FTIR and fluorescence spectroscopies, we demonstrate luminescence quenching of the decorated N_CNC by copper (II) sulfate, hypothesizing for these new functionalized materials an application in water purification technologies.

## 1. Introduction

The nanostructures isolated from native cellulose, such as cellulose nanofibers (CNFs), cellulose nanocrystals (CNCs) and bacterial cellulose (BC) [1,2,3], have recently attracted tremendous interest, thanks to their outstanding chemical and mechanical properties [4,5,6,7,8]. These nanomaterials of biologic origin are very appealing for numerous applications and have raised the industrial interest, since cellulose is abundant and can be isolated in crystalline nanostructures with low environmental impact [9]. Besides, nanocelluloses are colorless or even transparent and harmless for human health [10,11]. Their potential in industrial application is mainly related to their sustainable nature, which makes them good candidates as substitutes of oil derivatives for the manufacture of goods, in order to reduce pollution and better commit to the principles of green and blue economies [2]. The current research stream is exploring their use in high-volume industrial applications, such as in the fields of water treatment, in the paint and coating industry, in the building industry, in the hygiene sector and in the paper industry [12].

Siding the industrially oriented research, new emerging fields of application of nanocellulose include hydrogels and aerogels [13,14], emulsion stabilizers [15], biocatalyst immobilizers [16], biosensors [17], drug delivery systems [18], adsorbents for contaminants [19,20], nanocomposites for environmental remediation [21], photonic films and transparent substrates for optoelectronic devices, as well as new nanostructured electroactive materials [22,23,24,25,26].

Among the various typologies of nanocellulose, CNCs are defined, according to the standards of the Technical Association of Pulp and Paper Industry (TAPPI), as crystalline rods featuring a high aspect ratio, a diameter between 5 and 50 nm and a length ranging from 100 to 500 nm. CNCs are the smallest nanostructures which may be isolated from native cellulose and display a high content of crystalline domains. CNCs were discovered in the 1950s by Rånby and Battista [27,28], who independently isolated them by acid hydrolysis. However, only in the last 15 years was the high potentiality of cellulose nanocrystals re-evaluated in light of the new intriguing applications listed above.

CNCs display a high specific surface area (SSA) and expose pending hydroxyl groups on their surface: these chemical functionalities may be exploited to carry out surface functionalization of the nanocrystals. For this reason, the concept of topochemical functionalization of nanocellulose, i.e., a functionalization involving only the surface of the nanocrystals, has become crucial with respect to bulk functionalization, i.e., the complete dissolution of the cellulose crystalline structure due to complete derivatization of the cellulose polymer [29]. In this respect, only mild reaction protocols, with controlled reaction times, can prevent crystalline phase transitions or, even worse, dissolution of the cellulose polymer [30]. This approach sometimes brings some complications, such as the necessity to perform a sequence of chemical reactions on nanocellulose to convert the hydroxyl into an activated group, with the difficulty of obtaining a fine control over the surface functionalities, their number and homogeneity.

**Figure 1:**
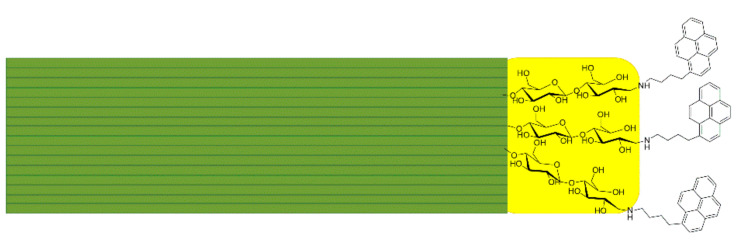
A cellulose nanorod, represented according to the model proposed in [32], with 1-pyrenyl units attached to the reducing ends (highlighted in yellow) by reductive amination.

An alternative and straightforward strategy for nanocellulose functionalization relies on a reaction used in polysaccharide technology, the reductive amination reaction [31], which modifies only the reducing ends of the cellulose nanocrystals, leaving the remaining nanocrystal surface unfunctionalized or available for further chemical manipulation. According to a model proposed so far [32], CNCs correspond to elementary fibrils and are rigid rods composed of 36 parallel cellulose polymer chains, kept together by intermolecular hydrogen bonds. The number 36 comes from the nature of the enzymatic complex which synthesizes cellulose in plants, the cellulose synthase (CESA) complex, composed by 6 rosettes each made of 6 enzymes which polymerize D-glucopyranose from UDP-glucose [33]. The trans-membrane enzymatic complex extrudes the 36 cellulose chains in parallel organization, and the chains associate into a cellulose I crystal structure [34] as soon as they leave the cell wall. Once cellulose nanocrystals have been recovered from a cellulose source by acid hydrolysis, they present the same crystalline association as in their native structure with the reducing termini of the polymer chains on the same side of the cellulose nanorod (as depicted in Figure 1).

The extensive functionalization of CNCs’ surface may drastically change their surface properties, for instance modifying their suspension-forming ability in water or the way they interact with oleophilic or oleophobic molecules. Conversely, an incomplete functionalization on the CNCs’ surface, for instance with a degree of substitution (DS) ≤0.37 [35], would not allow to understand the exact position of the new functional groups, since most of the reactions which may be performed on the cellulose polymer are only selective, but not specific towards the primary alcohol group [31]. This, for some applications, could be tricky. For this reason, an issue arises in which a mild reaction strategy allows specific functionalization of nanocellulose. In the present work, we use the reducing end functionalization to introduce 1-pyrenyl units as substituents on cellulose nanocrystals: the organic fluorophores are introduced to endow the nanocrystals with an additional property, i.e., pyrene-luminescence, while their hydrophilicity is preserved, being the pyrene units introduced only at the reducing end of the nanocrystal, as depicted in Figure 1. We demonstrate that pyrene-emission (and its excimer) is detectable in the functionalized nanocrystals in spite of the low amount of dye linked to the nanocrystals. Second, we demonstrate pyrene luminescence quenching in the presence of Cu(II) salts. These experiments demonstrate that, using the reductive amination reaction, it is possible to introduce an additional property on CNCs while leaving the remaining pending hydroxyl groups available for interaction with the solution or for further functionalization. In our view, this strategy is important in order to have the surface of the nanocrystal available for anchoring on filtration devices.

## 2. Results

### 2.1. Synthesis of the Pyrene Dye

**Scheme 1:**
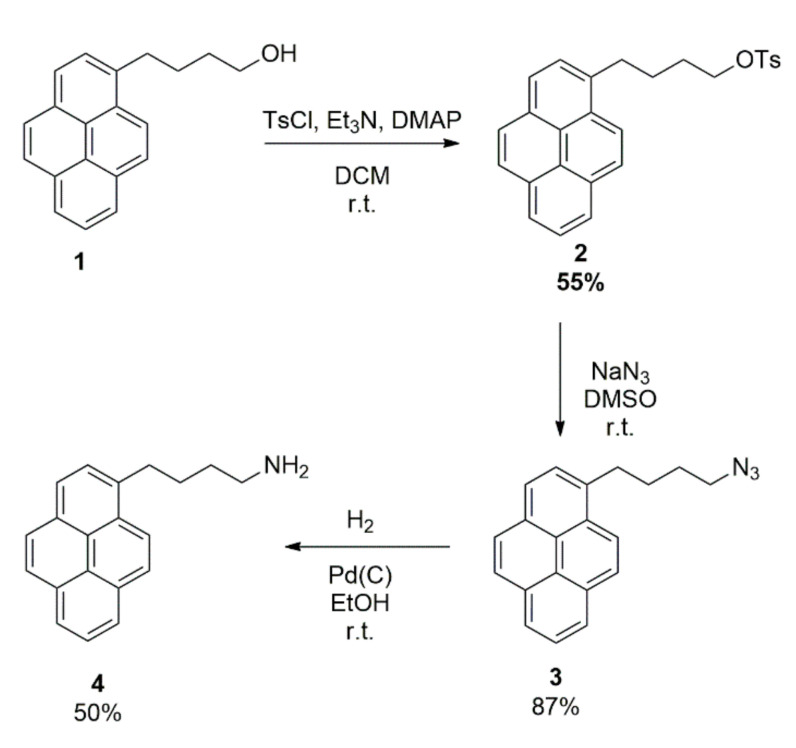
Synthetic sequence leading to 4-(1-pyrenyl)butanamine **4**.

For performing the reductive amination reaction, we needed a pyrene dye functionalized with a pending primary amine functionality. A good precursor of the 1-pyrenyl butanamine **4** was the commercially available 4-(1-pyrenyl)buthanol 1. The reaction sequence leading to compound **4** starting from **1** and the relative yields are reported in the Scheme 1. We first prepared the tosylate derivative **2** via a room temperature tosylation reaction of the alcohol group with a yield of 55%; following this, we converted the tosyl group into an azide by a nucleophilic substitution performed using sodium azide in DMSO at room temperature. The azide is a good precursor of primary amines, and was isolated in 87% yield. Finally, **4** was afforded in satisfactory yield (50%) by submitting the pyrene azide **3** to a hydrogenation reaction in the presence of palladium, supported on charcoal as a catalyst and EtOH as a solvent. The catalytic hydrogenation was much more efficient in furnishing **4** than the Staudinger reaction, which yielded a mixture of the primary amine with phosphine oxide. Our synthetic sequence slightly differs from other synthetic sequences reported by others [36,37], but allowed the isolation of **4** in good overall yield and good purity. The identity of **4** was assessed by ^1^H- and ^13^C-NMR spectroscopies and by LCMS-IT-TOF spectrometry (compare Figures S1–S6, Supplementary Materials).

### 2.2. Synthesis of Sulfated and Neutral CNCs

**Figure 2:**
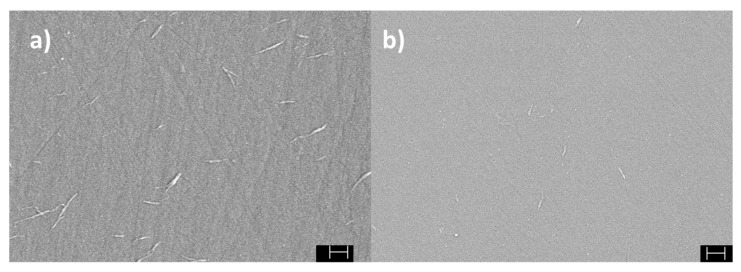
FE-SEM micrographs of (**a**) S_CNC deposited from 10 mg L^−1^ water suspension, and (**b**) N_CNC deposited from 1 mg L^−1^ DMSO suspension. Scale bar: 200 nm.

Cellulose nanocrystals were prepared by acid hydrolysis with either sulfuric acid or hydrochloric acid at controlled reaction conditions. The starting material was Avicel PH-10.1, a cotton linter-based commercial stationary phase for column chromatography. As extensively reported in the literature, sulfuric acid hydrolysis yields surface negatively charged S_CNC, due to a parallel process of surface sulfatation involving the primary hydroxyl groups on the surface glucopiranose units of the nanocrystals [38]. As explained in the next sections, the surface sulfatation determined aggregation of the cellulose nanocrystals in the presence of the salts used in the following experiments. For this reason, we also prepared neutral cellulose nanocrystals (neutral = no bonded surface charge) by hydrochloric acid hydrolysis from the same starting material. The uncharged nanocrystals were named N_CNC. N_CNC displayed a higher aggregation tendency in distilled water, as an effect of the absence of any surface charge. However, they could be characterized by FE-SEM microscopy by deposition from DMSO suspensions, in which they showed better stability. Figure 2 shows the FE-SEM micrographs recorded on (a) S_CNC deposited from water and (b) N_CNC deposited from DMSO on silicon substrates. Thanks to these microscopic observations, we could measure an average length of 149 ± 32 nm and 151 ± 17 nm for S_CNC and N_CNC respectively, while in both cases. the thickness was detected by AFM to be 10 ± 2 nm.

### 2.3. Synthesis of Pyrene Derivatives of CNCs

**Scheme 2:**
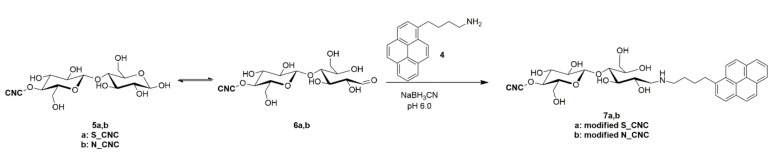
Reductive amination between **4** and sulfated or neutral cellulose nanocrystals.

The functionalization of cellulose nanocrystals with the dye **4** was conveniently performed in water solvent, dispersing the cellulose nanocrystals in a pH 6.0 and 50 mM phosphate buffer. A 1% molar ratio between the dye and the nanocellulose (mmol of **4** vs. mmol of glucopyranose) was used to perform the reaction. The functionalization was carried out in the presence of a molar excess of sodium cyanoborohydride. The synthesis of the pyrene-functionalized cellulose nanocrystals is depicted in Scheme 2. At the end of the reaction time, the nanocrystals were purified by washing with methanol, followed by dialysis against mQ water to eliminate traces of unreacted dye and excess salts. Finally, the samples were freeze-dried and used in the next experiments. An AFM check of the preserved nanocrystals’ morphology was performed on their thin films. Figure S7, reported in the Supplementary Materials, demonstrates the preserved nanorod aspect of the S_CNC sample after the reductive amination.

**Figure 3:**
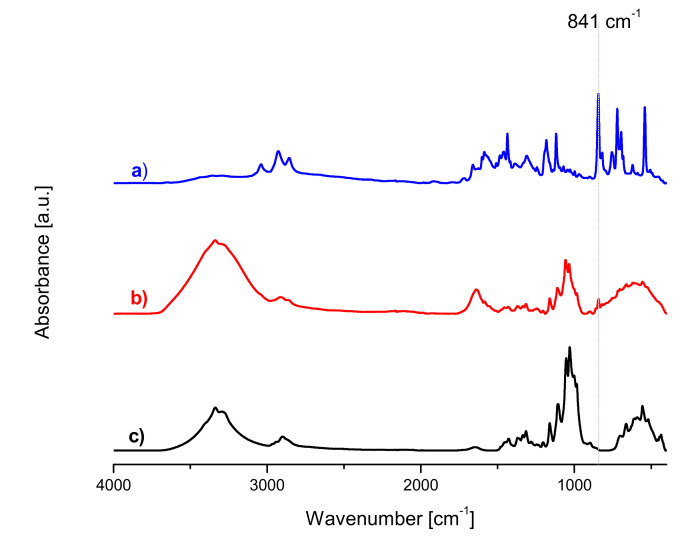
ATR-FTIR spectra of (**a**) 4-(1-pyrenyl)butanamine **4**, (**b**) pyrene-modified S_CNC **7a** and (**c**) unmodified S_CNC.

ATR-FTIR spectroscopy performed on the freeze-dried samples was very useful to have an indication of the success of the functionalization. Figure 3 shows the ATR-FTIR spectra of the 4-(1-pyrenyl)butanamine **4** (blue trace), of S_CNC (black trace) and of the modified S_CNC sample **7a** (red trace). The spectrum of the cellulose nanocrystals displayed the common features of cellulose I, with a strong absorption band at 3340 cm^−1^ attributed to -OH groups’ stretching, forming intramolecular hydrogen bonds. Absorption bands attributed to C-H stretching modes below 3000 cm^−1^ were also clearly visible and centered in the nanocelluloses at 2900 cm^−1^. The signal centered at 1644 cm^−1^ in CNCs could be attributed to residual water contained in the cellulose nanocrystals. The intense band between 920 and 1220 cm^−1^ in the nanocrystals was attributed to stretching modes of single C-O-C bonds of the acetal units.

Butanamine **4** presented distinctive signals, such as a broad absorption at ~3350 cm^−1^ (stretching of -NH_2_ group), characteristics of absorption of aromatic C-H stretching at 3044 cm^−1^, -NH_2_ group bending modes at ~1574 and in the region below 900 cm^−1^, besides aromatic stretching modes in the region between 1600 and 1400 cm^−1^ and intense aromatic C-H bending modes below 900 cm^−1^. In particular, the intense absorption band centered at 841 cm^−1^ could be attributed to aromatic C-H bending and produced a small overtone at ~1670 cm^−1^.

The modified S_CNC **7a** showed typical features of the cellulose nanocrystals in their spectrum, such as the broad absorption band attributed to the -OH stretching, but with the appearance of features representative of the butanamine **4**. First, the increase in intensity of the -OH stretching band with respect to the acetal stretching band pointed at an increase in the sample of hydrogen bond-donating groups (hence the presence of secondary amine functionalities and additional hydroxyl groups in the reducing termini introduced by the reaction), with the contemporary loss in intensity of acetal absorption due to the reducing ends turned from hemiacetal into secondary amines. The broad band of -OH stretching also presented a small shoulder corresponding to the resonance frequency of aromatic C-H stretching at 3044 cm^−1^. Presence of the aromatic units was also evident from the increase in intensity of the band attributed to crystallized water, overlapped to the overtone of the bending absorption of aromatic C-H. Finally, the strong absorption at 841 cm^−1^, attributed to aromatic C-H bending, appeared as a distinctive absorption in the spectrum of modified S_CNC **7a**.

### 2.4. Photophysical Characterization

**Figure 4:**
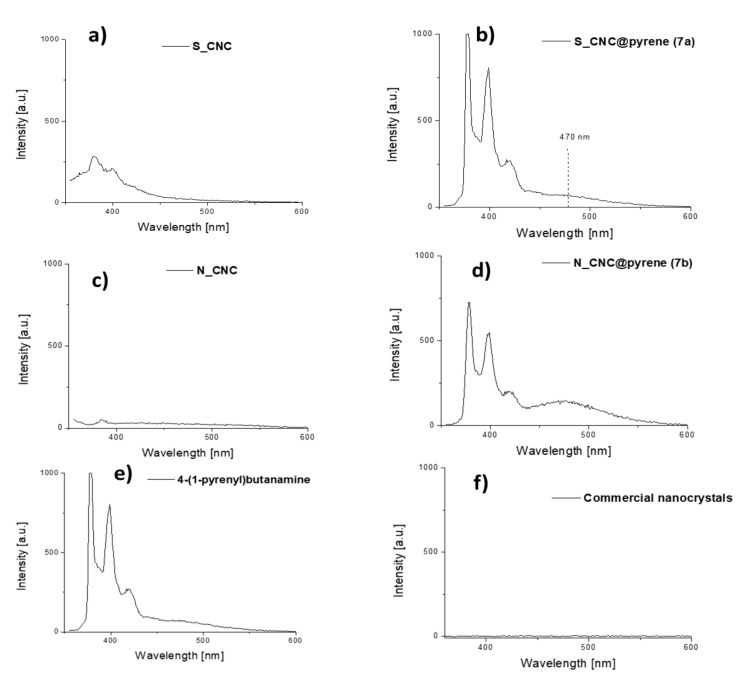
Emission spectra of cellulose nanocrystals recorded in 0.2 mg mL^−1^ DMSO suspensions: (**a**) S_CNC, (**b**) pyrene-modified S_CNC **7a**, (**c**) N_CNC, (**d**) pyrene-modified N_CNC **7b**, (**e**) spectrum of 4-(1-pyrenyl)butanamine **4** in the same solvent and (**f**) commercial sulfated CNCs. Excitation wavelength = 345 nm. Emission spectra of S_CNC and N_CNC were recorded using a 1 cm cuvette. Emission spectra of pyrene-modified S_CNC and N_CNC **7a** and **7b** were recorded using a 0.1 cm cuvette.

Emission spectra of the pristine S_CNC and N_CNC and of the pyrene-modified nanocrystals **7a** and **7b** are shown in Figure 4, while the positions of the emission maxima are listed in Table 1. Emission spectra were collected in DMSO to evidence the presence or not of the excimer of pyrene, whose emission is centered at ~470 nm. The pyrene butanamine **4** showed a distinctive emission profile, with characteristic emission maxima at 378, 399, 420 and 475 nm (Figure 4e). The last emission peak was absent in water solution and detectable in DMSO (compare Supplementary Figure S8 for the relevant emission spectrum of **4** in water). As expected, modified S_CNC and N_CNC **7a** and **7b** showed the distinctive emission peaks of butanamine **4** at the following wavelengths: 378, 398, 419 and 470 nm. Surprisingly, both pristine S_CNC and N_CNC showed an emission in the UV region, upon excitation at 345 nm, with considerably lower intensity than the pyrene-modified nanocrystals **7a** and **7b**. This emission was not observed in commercial sulfated cellulose nanocrystals (panel f, Figure 4), which were investigated as a control sample. As it will be explained in the Discussion Section, this phenomenon was attributed to the cellulose source used for the preparation of cellulose nanocrystals by acid hydrolysis.

### 2.5. Luminescence Quenching Experiments

**Figure 5:**
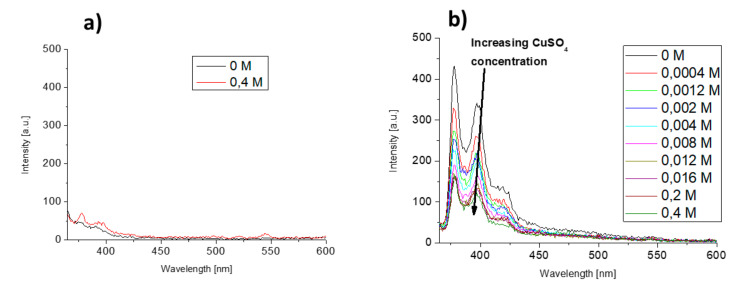
Emission spectra of N_CNC recorded in 0.2 mg mL^−1^ water solutions in the presence of Cu^2+^ salts: (**a**) pristine N_CNC and (**b**) pyrene-modified N_CNC **7b**. The insets show the molar concentration of salts used.

Luminescence quenching experiments were investigated on both pyrene-modified S_CNC and N_CNC **7a** and **7b** in the presence of increasing concentration of diverse salts in water suspensions. The scope of the experiments was to have a preliminary information on whether the pyrene-modified cellulose nanocrystals interacted preferentially with some salts and were therefore suitable to develop sensing systems in water or to being immobilized in membranes for water purification. Several salts were tested: NaCl, FeCl_3_, CuSO_4_ · 5H_2_O, Hg(OAc)_2_. The Figure 5 shows the result of CuSO_4_ slow addition to a water suspension of N_CNC (left panel) and pyrene-modified N_CNC 7b (right panel). The same experiments were performed on S_CNC, but the pyrene-modified S_CNC 7a yielded inconsistent response to the addition of each salt (data not shown in this paper), due to aggregation and precipitation of the nanocrystals. Pyrene-modified N_CNC 7b, instead, yielded a rational response only in the presence of copper sulfate, while were apparently unperturbed by the other salts. These results will be better discussed in the next paragraph.

## 3. Discussion

The reductive amination reaction allowed us to carry out a covalent functionalization of the cellulose nanocrystals with 4-(1-pyrenylbutanamine) **4**, availing of a very mild reaction protocol performed in water. Purification of the sample was straightforward, as it was performed by dialysis in water. To assess the effectiveness of the functionalization, we acquired the ATR-FTIR spectra of S_CNC and their pyrene-modified derivative **7a**, identifying the signals of the pyrene aromatic system in the spectrum of the hybrid system. In order to confirm the effectiveness of the reaction, we also coupled an aliphatic amine with D-glucopyranose in the same conditions, successfully isolating the corresponding amination product (data not shown in this paper). This allowed us to understand that the proposed synthetic protocol is reliable for the effective conversion of the imine intermediate (the Schiff’s base generated as a result of the nucleophile attack of the primary amine to the aldose unit) into a secondary amine by sodium cyanoborohydride. The amount of dye bonded to the nanocrystals was very low, as the reductive amination reaction was performed with a molar ratio of **4**:glucopiranose units of 1:100, and the linked pyrene dyes were not detectable by UV-Vis spectroscopy, as they were covered by the scattering produced by the nanocrystals (see Supplementary Figures S10 and S11 acquired for the S_CNC samples).

Then, we started the luminescence characterization. Unexpectedly, recording the reference spectra for S_CNC and N_CNC, we found an emission from both samples, upon excitation of the relevant suspensions at the wavelength of 345 nm, that peaked at 378 and 400 nm for S_CNC and 383 nm for N_CNC (compare Table 1). The relevant spectra for DMSO suspensions are the ones reported in Figure 4a,c. This emission was found on spectra recorded in DMSO and in water (for the relevant emission spectrum recorded from N_CNC water suspensions, please refer to Figure 5a, black trace). Suspecting a possible contamination of the products during their preparation in our laboratories, we repeated the preparation of both typologies of cellulose nanocrystals, by hydrolysis with sulfuric acid or hydrochloric acid, several times in the same and also in different laboratories, each time recording the same result. To finally understand the possible origin of this emission from our cellulose nanocrystals, in the same conditions, we analyzed commercial CNCs purchased from CelluForce. The relevant emission spectrum is reported in Figure 4f. The commercial cellulose nanocrystals did not show any emission feature. Commercial CNCs from cellulose are produced, as declared by the company, by sulfuric acid hydrolysis from wood pulp. Hence, we started to hypothesize whether the emission from the CNCs produced in our laboratories was dependent on the nature of the starting cellulose material, the microcrystalline cellulose Avicel PH-10.1. This microcrystalline cellulose derives from cotton by hydrolysis with diluted mineral acids [39], has a high cellulose I content with very high crystallinity index and it is often used as a reference for XRD or Raman assessment of the crystallinity index of cellulose [40,41].

The luminescent properties of cellulose are the subject of controversial literature reports. Actually, our CNCs displayed a similar emission to the one observed from Avicel by Castellan et al. [42], attributed to heteroaromatics of the furan and pyron type, possibly formed in traces by degradation of the polysaccharide chain. A similar hypothesis was formed by Kalita et al. [43], who attributed the autofluorescence at ~400 nm found in their synthetic CNCs to the presence of fluorescent subunits identified in the FTIR spectra as lignin fragments, such as *p*-coumeric and ferulic acid or phenolic structures. Ding et al. [44] attributed the autofluorescence of cellulose to a partial double-bond character displayed by the glycosidic bond, whose presence was also hypothesized in disaccharides such as cellobiose and maltose. However, it was demonstrated by Arndt and Stevens [45], using UV-circular dichroism, that mono- and di-saccharides absorb only between 150 and 190 nm. A final interpretation was that by Gan et al. [46], who hypothesized that the emission recorded from cellulose nanocrystals was the consequence of a Stokes scattering [47,48] enhanced by CNC-oriented assembly [49]. In our case, it is very difficult to understand which can be the origin of the luminescence observed in CNCs, as the FTIR spectra of our pristine CNCs also did not reveal the presence of aromatic structures. However, the native emission intensity was very low if compared to the luminescence of pyrene-modified nanocrystals, and it was not responsive to the titration experiments that we performed with salts (compare Figure 5a).

Pyrene-modified S_CNC and N_CNC **7a–b** displayed the distinctive emission features of 4-(1-pyrenyl)butanamine **4**, with emission peaks at 378, 398, 419 and 470 nm. This result was remarkable considering the very low amount of dye used during the functionalization reaction (1 molar % with respect to glucopyranose units). The emission wavelength of 470 nm observed in DMSO was attributed to pyrene excimer formation. At this point, we decided to investigate how interaction with cations influenced the emission intensity from the cellulose nanocrystals, with the aim to understand whether a luminescence quenching induced by any of the salts used could suggest a possible application of the systems that we had prepared in water purification technology. During this investigation, we had to discard experiments conducted on pyrene-modified S_CNC **7a**, because interaction with cations of these surface-negatively charged nanorods induced their aggregation, preventing any reasonable conclusion on their luminescence behavior. We took this behavior as a demonstration that the terminal functionalization had left the surface of the S_CNC unperturbed, exposed to the external solution and therefore free to interact with the other solutes. Conversely, Zhang et al. [50] reported an extensive surface functionalization of S_CNC with pyrene units, and no aggregation was observed in the presence of a wide number of cations. Pyrene-modified N_CNC **7b** yielded a luminescence quenching response only in the presence of increasing concentrations of copper sulfate, while it was apparently unperturbed by NaCl and Hg(OAc)_2_. FeCl_3_ was discarded as a quencher, since it displayed an absorption in the relevant region for the experiments (overlapping absorption of the pyrene dye). Thus, among the salts investigated, the pyrene-modified N_CNC **7b** selectively interacted only with copper sulfate.

The presence of two vicinal -OH and secondary amine groups, formed on the C1 and C2 positions of the terminal polysaccharide unit of the nanocrystal in the pyrene-modified N_CNC **7b**, could potentially act as a bidentate ligand for metal cations, with potential assistance from other neighboring -OH groups, while the free -OH pending groups on the CNC surface remain available for establishing H-bonds, which could be, for instance, used to anchor the nanocrystals on a surface or on paper, to obtain a sustainable filtration device. A similar principle was exploited by Lu et al. [51] to prepare a hydrophilic fluorescent paper sensor for nitroaromatic compounds (NACs) exploiting pyrene sensing ability, towards whom our pyrene-cellulose derivatives could be exploited as well.

## 4. Materials and Methods

### 4.1. Synthesis of 4-(1-pyrenyl)Butanamine ***4***

**General:** All reagents were purchased at the highest commercial quality and used without further purification. Reactions were carried out under a nitrogen atmosphere in oven-dried glassware, using dry solvents unless otherwise stated. Dichloromethane was distilled immediately prior to use on phosphorus pentoxide. Anhydrous grade dimethylsulfoxide was used and dispensed under nitrogen. Absolute ethanol was used without further purification. Preparative column chromatography was performed using silica gel 60, particle size 40 ÷ 63 μm from Merck. Merck silica gel 60 F254 aluminium sheets were used for TLC analyses. All new compounds were characterized by ^1^H-NMR, ^13^C-NMR, FTIR spectroscopy and HMRS spectrometry. GC-MS analyses were performed on a gas chromatograph equipped with an SE-30 (methyl silicone, 30 m × 0.25 mm id) capillary column and an ion trap selective mass detector. ^1^H-NMR and ^13^C-NMR spectra were recorded at 500 MHz and 125 MHz, respectively, on a Bruker Avance AM 500, using the residual proton peak of CDCl_3_ at 7.26 ppm as reference for ^1^H spectra and the signals of CDCl_3_ at 77 ppm for ^13^C spectra. Coupling constants values are given in hertz. High-resolution mass spectra were acquired on an Agilent high performance liquid chromatography-QTOF spectrometer via direct infusion of the samples using methanol or water as the elution solvent. Melting points (uncorrected) were determined on a Stuart Scientific Melting point apparatus SMP3.

**4-(1-pyrenyl)butyl tosylate 2:** A 2 neck round bottom flask was charged under nitrogen with 0.5 g of 4-(1-pyrenyl)buthanol (1.8 mmol), 0.22 g of dimethylaminopyridine (1.8 mmol), 5 mL of dry dichloromethane and 0.5 mL of triethylamine (3.6 mmol). The system was cooled to 0 °C and a solution of tosyl chloride (0.7 g, 3.6 mmol in 5 mL) was added dropwise. The system was kept at 0 °C for 2 h, then it was allowed to warm to room temperature and stirred until TLC analysis revealed complete disappearance of the starting material. The reaction was quenched with 30 mL of HCl 1.5 N. The mixture was extracted with dichloromethane (3 × 30 mL). The organic extracts were collected and dried over anhydrous Na_2_SO_4_, filtered and the solvent was distilled under reduced pressure. The crude material was purified by column chromatography on silica gel using hexane and ethyl acetate in volumetric ratio 8:2 as the eluent. 0.423 g of a yellow wax were isolated (yield 55%). ^1^H-NMR (CDCl_3_, 500 MHz): δ8.23-8.15 (m, 3H), 8.10 (d, 2H, *J* = 8.1 Hz), 8.03 (s, 2H), 8.01 (t, 1H, *J* = 8.1 Hz), 7.80 (d, 2H, *J* = 7.8 Hz), 7.76 (d, 2H, *J* = 7.8 Hz), 7.25 (d, 2H, *J* = 7.3 Hz), 4.09 (t, 2H, *J* = 6.2 Hz), 3.31 (t, 2H, *J* = 7.5 Hz), 2.36 (s, 3H), 1.98–1.72 (m, 2H) ppm. ^13^C-NMR (CDCl_3_, 126 MHz): δ144.6, 135.8, 133.10, 131.4, 130.8, 129.9, 129.8, 128.6, 127.8, 127.5, 127.3, 127.15, 126.7, 125.9, 125.10, 125.0, 124.9, 124.8, 123.14, 70.3, 32.7, 28.7, 27.5, 21.5, 14.19 ppm.

**1-(4-azidobutyl)pyrene 3:** A 100 mL round bottom flask was charged with 0.4 g of 4-(1-pyrenyl)butyl tosylate (0.93 mmol), 0.091 g of sodium azide and 5 mL of dry DMSO under nitrogen atmosphere. The mixture was stirred at room temperature for one night. After this time TLC analysis revealed the complete disappearance of the starting material. The mixture was diluited with 30 mL of brine and extracted with ethyl acetate (3 × 30 mL). The organic extracts were collected and washed with brine (2 × 30 mL), dried over anhydrous Na_2_SO_4_, filtered and the solvent was distilled under reduced pressure. 0.241 g of a white solid were isolated (yield 87%). Mp = 78–79 °C. ^1^H-NMR (CDCl_3_, 500 MHz): δ8.26 (d, 1H, *J* = 9.5 Hz), 8.17 (dd, 2H, *J*_1_ = 7.5, *J*_2_ = 4.0 Hz), 8.12 (d, 2H, *J* = 8.5 Hz), 8.04 (d, 1H, *J* = 9.5 Hz), 8.02 (d, 1H, *J* = 9.5 Hz), 7.99 (d, 1H, *J* = 7.5 Hz), 7.86 (d, 1H, *J* = 8.0 Hz), 3.38 (t, 2H, *J* = 7.5), 3.34 (t, 2H, *J* = 7.5 Hz), 1.96 (quintuplet, 2H, *J* = 7.5 Hz), 1.78 (quintuplet, 2H, *J* = 7.5 Hz). ppm. ^13^C-NMR (CDCl_3_, 126 MHz): δ136.10, 131.4, 130.9, 129.9, 128.6, 127.5, 127.3, 127.18, 126.7, 125.8, 125.10, 125.0, 124.9, 124.8, 124.7, 123.18, 51.4, 33.0, 28.9, 28.8 ppm.

**4-(1-pyrenyl)butanamine 4:** A 250 mL round bottom flask was charged with 0.22 g of 1-(4-azidobutyl)pyrene (0.74 mmol), 0.079 g of Pd(C) (10%) (0.074 mmol) and 15 mL of absolute ethanol. The mixture was degassed by nitrogen bubbling for 10 min. Afterwards hydrogen was blown into the mixture using a syringe and a balloon. The mixture was stirred at the room temperature for 48 h. After this time, TLC analysis revealed complete disappearance of the starting material. The solvent was removed under reduced pressure and the crude material was purified by column chromatography on silica gel using dichloromethane, methanol and triethylamine in volumetric ratio 90:10:1 as the eluent. 100 mg of a yellow wax was isolated (yield 50%). ^1^H-NMR (CDCl_3_, 500 MHz): δ8.26 (d, 1H, *J* = 8.3 Hz), 8.16 (dd, 2H, *J*_1_ = 8.16 Hz, *J*_2_ = 4.0 Hz), 8.10 (dd, 2H, *J*_1_ = 8.10 Hz, *J*_2_ = 3.0 Hz), 8.06–7.93 (m, 3H), 7.86 (d, 1H, *J* = 7.86 Hz), 3.36 (t, 2H, *J* = 8.0 Hz), 2.76 (t, 2H, *J* = 7.0 Hz), 1.89 (quintuplet, 2H, *J* = 8.0 Hz), 1.58 (m, 2H) ppm. ^13^C-NMR (CDCl_3_, 126 MHz): δ136.8, 131.4, 130.9, 129.8, 128.6, 127.5, 127.2, 127.17, 126.5, 125.8, 125.10, 125.0, 124.8 (two signals), 124.6, 123.4, 42.10, 33.7, 33.3, 29.13 ppm. LCMS-IT-TOF calculated for C_20_H_19_N: 273.1517, found (M + H)^+^: m/z 274.1588.

### 4.2. Hydrolysis of Cellulose, Nanocrystals Isolation and Functionalization

**General** Avicel PH-101 was used as the starting material for nanocrystalline cellulose isolation. Sonication of nanocellulose suspensions was carried out with a Branson Sonifier 250 (Danbury, CT, USA). Dialysis was carried out at room temperature against mQ water in nitrocellulose tubes with a cut off of 12,600 Da. Commercial nanocrystals used for acquisition of the emission spectrum in the Figure 4f were a donation from the Pharmacy Department of the University of Pisa, and had been purchased from CelluForce.

**Synthesis of sulfated CNCs 5a:** This procedure was adapted from Operamolla et al. [52] 47 mL of deionized water were introduced in a 250 mL three necked round-bottom flask equipped with a water condenser and a mechanical stirrer. Then, the flask was cooled in an ice bath and 47 mL of concentrated H_2_SO_4_ were added. After that, 5 g of Avicel PH-101 were added and the suspension was warmed to 50 °C for 80 min. The system was cooled to room temperature and the mixture was transferred to polypropylene centrifugation tubes. Centrifugation at 4000 rpm was repeated replacing the supernatant solution with fresh deionized water until the pH was approximately 1. Then, the precipitate was suspended in deionized water with the aid of a Branson sonifier 250 (Danbury, CT, USA) equipped with an ultrasonic horn with 3.5 mm diameter (micro tip) operated in pulsed mode, with a power of 40 W, 0.6 s pulses for 10 min and dialyzed against distilled water until neutrality using a cellulose nitrate membrane with a molecular weight cut-off of 12,400 Da. The resulting suspension was transferred to polypropylene centrifugation tubes and centrifuged at 4000 rpm for 20 min. The supernatant solution was kept and water was removed under reduced pressure, yielding 925 mg of cellulose nanocrystals with an average length of 280 ± 70 nm.

**Synthesis of neutral CNCs 5b:** 50 mL of deionized water were introduced in a 250 mL three necked round-bottom flask equipped with a water condenser and a mechanical stirrer. Then, the flask was cooled in an ice bath and 50 mL of concentrated HCl were added. After that, 5 g of Avicel PH-101 were added and the suspension was warmed to 105 °C for 6 h. The system was cooled to room temperature, diluted with 50 mL of distilled water and the mixture was transferred to polypropylene centrifugation tubes. Centrifugation at 1000 rpm for 10 min was repeated 4 times replacing the supernatant solution with fresh deionized water until the pH was approximately 3. Then the precipitate was dialyzed against distilled water until neutrality using a cellulose nitrate membrane with a molecular weight cut-off of 12,400 Da. The resulting suspension was recovered and water was removed by distillation under reduced pressure, yielding 4.713 g of cellulose nanocrystals.

**General Procedure for the reductive amination of cellulose nanocrystals (7a,b):** In a 100 mL round bottom flask 500 mg of cellulose nanocrystals (3.1 mmol) were suspended in a 50mM phosphate buffer (pH = 6, 50 mL) using Branson sonifier 250 (Danbury, CT, USA) equipped with an ultrasonic horn with 3.5 mm diameter (micro tip) operated in pulsed mode, with a power of 40 W, 0.6 s pulses for 10 min in the case of sulfated CNCs and operated in constant mode in the case of neutral CNCs. A solution of 4-(1-pyrenyl)butanamine (8 mg, 0.03 mmol) in phosphate buffer (5 mL) was added, followed by 100 mg of NaBH_3_CN (14.6 mmol). The mixture was stirred at room temperature for 72 h. After this time, the mixture was diluted with methanol (50 mL) and centrifuged at 4000 rpm for 10 min. The liquid phase was removed and replaced with fresh methanol, the systems were mixed and centrifuged again at 4000 rpm for 10 min. The washing operation was repeated two more times. Then, the precipitate was resuspended in water (30 mL) and dialyzed against distilled water using a cellulose nitrate membrane with a molecular weight cut-off of 12,400 Da. Water was distilled under reduced pressure and a white solid was isolated (0.286 g, 57% yield in the case of sulfated CNCs; quantitative yield in the case of neutral CNCs).

### 4.3. Characterization

**ATR-FTIR Analyses** FTIR spectra were recorded on a Spectrum, Perkin-Elmer, Waltham, MA, USA. Two spectrophotometer equipped with an UATR Accessory. The nanocellulose powders were placed in direct contact with the diamond/ZnSe crystal without the aid of any solvent. For the same analysis on 4-(1-pyrenyl)butanamine, a drop of chloroform solution with the concentration of 1 mg/mL was deposited on the crystal and the solvent was allowed to evaporate at room temperature before the measurement was acquired.

**FE-SEM images** The morphology of the surface of nanocrystalline cellulose was analyzed by FE-SEM, on a Field Emission Scanning Electron Microscope, ZEISS Merlin, Jena, Germany, equipped with a GEMINI IIs column and Beam-Booster, with acceleration voltages between 0.05 and 30 kV and 0.8 nm as the best resolution, four optional detectors for SE and BSE, charge compensation and an in situ sample cleaning system. Silicon slabs were used as substrates for FE-SEM measurements. For S_CNC investigation, a solution 0.01% by weight of nanocrystals in water was drop cast on a silicon slab and allowed to dry overnight. For N_CNC investigation, a solution 0.001% by weight of nanocrystals in DMSO was drop cast on a silicon slab and allowed to dry overnight.

**AFM Microscopy** Atomic force microscopy topographies (Figure S6) were taken using a XE-100 SPM, Park, Suwon, Korea, system microscope. Images were acquired in the tapping mode using tips (Type PPP-NCHR) on a cantilever of 125 μm length, about 330 kHz resonance frequency, 42 N m^−1^ nominal force constant and 10 nm guaranteed tip curvature radius. Surface areas were sampled with a scan rate of 1 Hz. The topographies were analysed using the software XEI (Park System Corporation, version 1.8.0).

**UV-VIS Spectroscopy** Analyses were recorded on a Spectrophotometer, Shimadzu, Kyoto, Japan, using the software Spectrum, with 1 cm cuvettes in distilled water or spectrophotometric grade DMSO. Cellulose nanocrystals were analyzed at concentration of 0.2 mg/mL.

**Emission Spectroscopy** Fluorescence spectra were recorded on Cary Eclipse Instrument, Agilent, Santa Clara, CA, USA, with the software Scan. Cellulose nanocrystals were analysed at a concentration of 0.2 mg/mL, in 0.1 cm cuvettes. Titration experiments with heavy metal salts were performed adding increasing volumes of a mother solution of the salt at a concentration of 100 mg/mL in distilled water.

## 5. Conclusions

In this paper, we have shown how the reductive amination reaction can be used as an effective synthetic protocol to covalently bind luminescent dyes to cellulose nanocrystals. Pyrene-modified S_CNC and N_CNC **7a,b** displayed the emission profile of 4-(1-pyrenyl) butanamine **4**, with emission peaks at 378, 398, 419 and 470 nm, though a very low amount of dye was used during the functionalization reaction (1 molar % with respect to glucopyranose units). The luminescence behavior of the modified nanocrystals, under excitation at the wavelength of 345 nm, was compared with the behavior of the pristine cellulose nanocrystals, evidencing a low emission from nanocellulose, centered at around 400 nm, whose origin is controversial and under literature debate. However, the emission properties of the pyrene-modified samples were clearly identified, and we could test the effect of interactions with different salts on the luminescence properties. In our experiments, N_CNC were revealed to be best-suited to investigate interaction with salts, as they lack any surface-negatively charged group which could induce nanocrystals’ aggregation in the presence of positive cations. Pyrene-modified N_CNC **7b** showed luminescence quenching in the presence of copper sulfate, and this suggests a possible application of these systems in water purification technologies, especially after their immobilization on sustainable devices composed of biodegradable cellulose paper.

## Figures and Tables

**Table 1:** Emission maxima of all samples measured in DMSO.

Sample	λ_em,DMSO_ (nm)
**4**	378, 399, 420, 475 ^1^
S_CNC	378, 400
N_CNC	383
Commercial sulfated nanocrystals	-
Pyrene-modified S_CNC **7a**	378, 398, 419, 470
Pyrene-modified N_CNC **7b**	378, 398, 419, 470

^1^ Emission peaks of 4-(1-pyrenyl)butanamine **4** in water: 378, 399, 420 nm (Supplementary, Figure S8). Relevant absorption spectra of **4** in DMSO, CHCl_3_ and water are reported in the Supplementary, Figures S9 and S10. UV-Vis Absorption spectra of S_CNC and pyrene-modified S_CNC 7a are reported in the Supplementary, Figures S11 and S12.

## Data Availability

Data are contained in the article and Supplementary Materials.

## Supplementary Materials

The following are available online, Figures S1–S12: ^1^H-NMR and ^13^C-NMR spectra of compounds **2**, **3** and **4**, AFM topography of a pyrene-modified S_CNC **7a** film, emission of **4** in water, UV-Vis absorption profiles of 4-(1-pyrenylbutanamine) **4** in chloroform, DMSO and water solution, UV-Vis absorption profile of S_CNC and pyrene-modified S_CNC **7a** in DMSO suspension.

## Sample Availability

Samples of the compounds shown in this paper are available upon request from the corresponding author.
