# Supplementary material for: Reductive Amination Reaction for the Functionalization of Cellulose Nanocrystals
Source: Molecules. 2021 Aug 19;26(16):5032. doi: 10.3390/molecules26165032 (PMC8400508; doi:10.3390/molecules26165032)
Supplement: Supplementary file 1 [file molecules-26-05032-s001.zip › molecules-1299225-supplementary.pdf]

molecules

Supplementary Material

Reductive Amination Reaction for the Functionalization of Cellulose Nanocrystals

Omar Hassan Omar [1], Rosa Giannelli [2], Erica Colaprico, [2] Laura Capodieci, [3] Francesco Babudri, [2] and Alessandra Operamolla [4,*]

[1] CNR-ICCOM Istituto di Chimica dei Composti Organometallici, Via Edoardo Orabona 4, 70126 Bari, Italy; hassan@ba.iccom.cnr.it
[2] Dipartimento di Chimica, Università degli Studi di Bari Aldo Moro, Via Edoardo Orabona 4, 70126 Bari, Italy; francesco.babudri@uniba.it
[3] ENEA – Italian National Agency for New Technologies, Energy and Sustainable Economic Development, Laboratory for Functional Materials and Technologies for Sustainable Applications (SSPT-PROMAS-MATAS), S.S. 7 "Appia" km 706, I-72100 Brindisi (Br), Italy; laura.capodieci@enea.it
[4] Dipartimento di Chimica e Chimica Industriale, Università di Pisa, Via Giuseppe Moruzzi 13, 56124 Pisa, Italy
* Correspondence: alessandra.operamolla@unipi.it; Tel.: 0039 050 2219342.

.

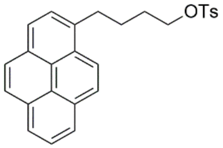

2

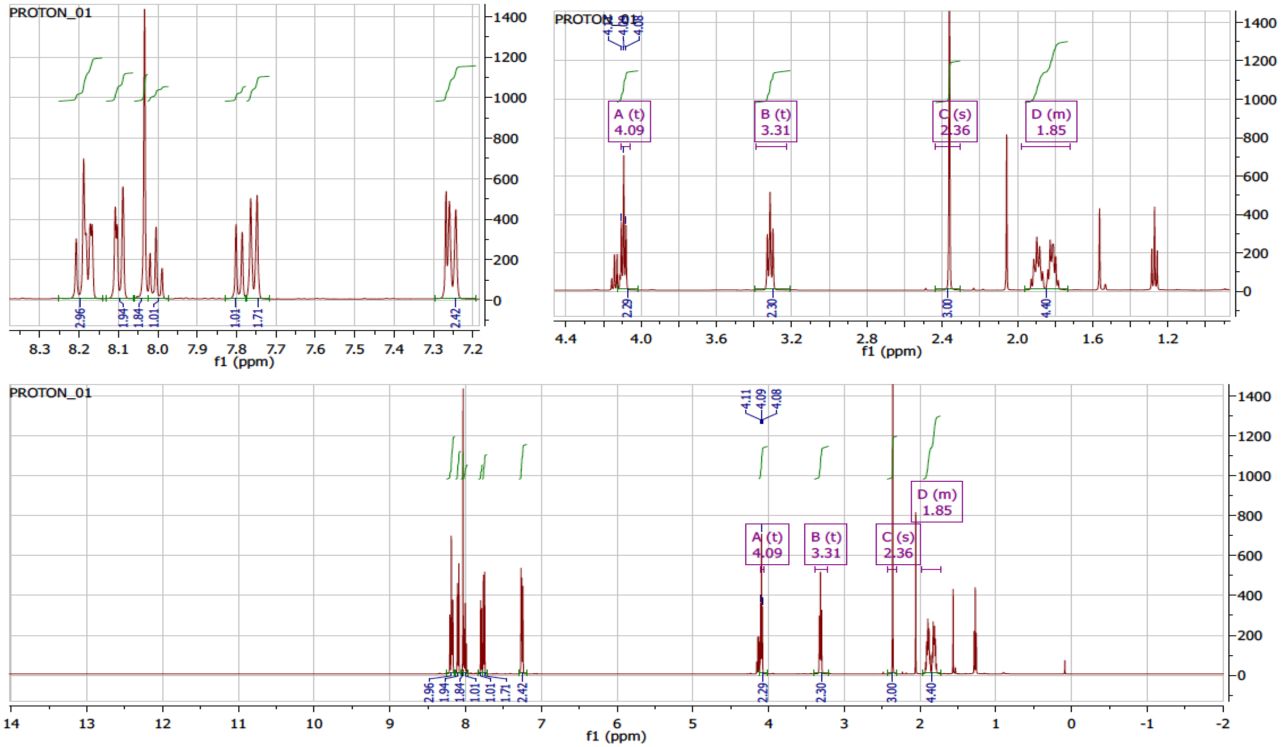

Figure S1. ^{1}H-NMR spectrum of 4-(1-pyrenyl)butyl tosylate **2** in $CDCl_3$.

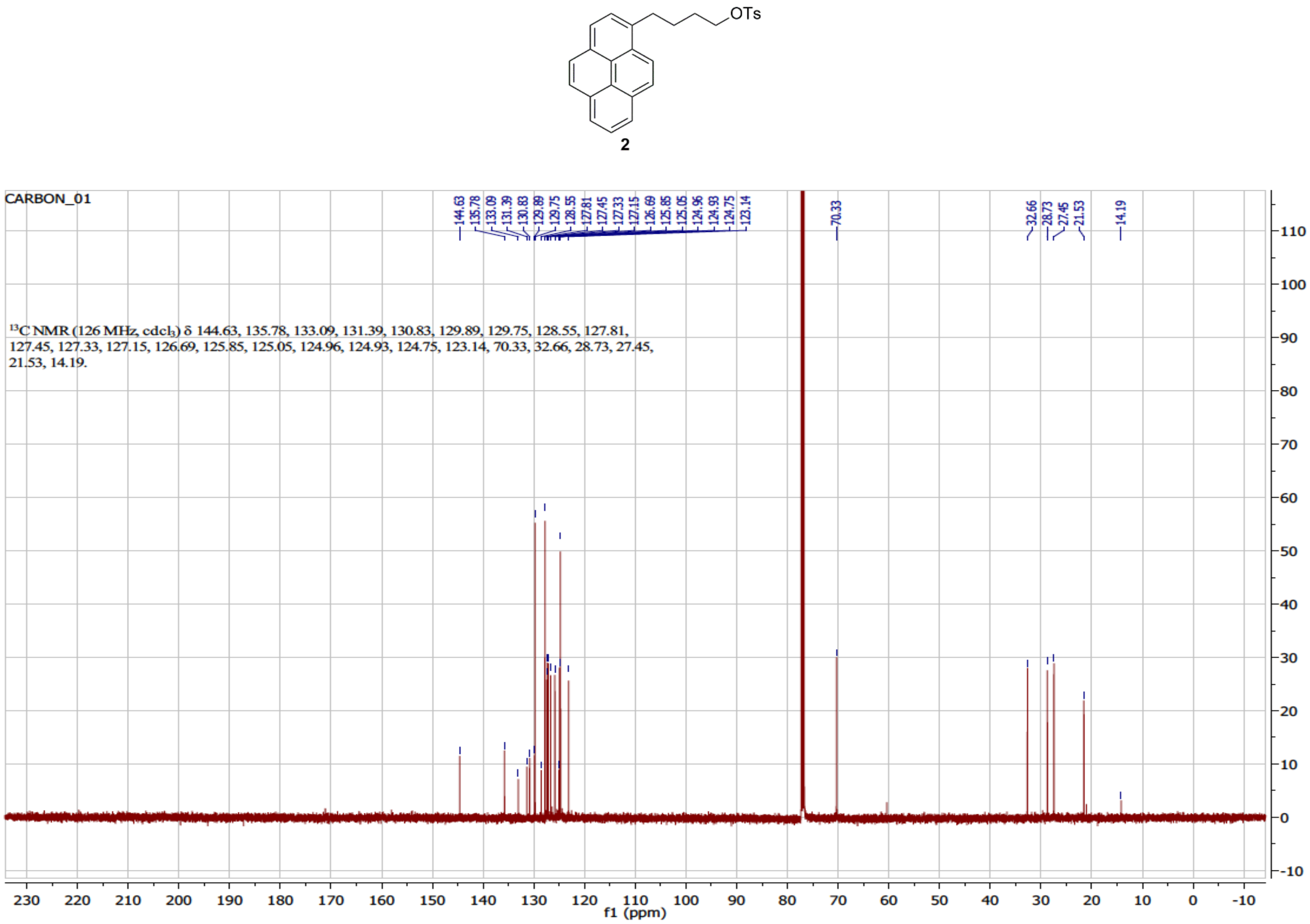

Figure S2. ^{13}C-NMR spectrum of 4-(1-pyrenyl)butyl tosylate **2** in $CDCl_3$.

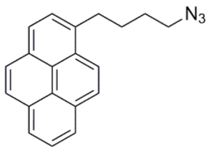

3

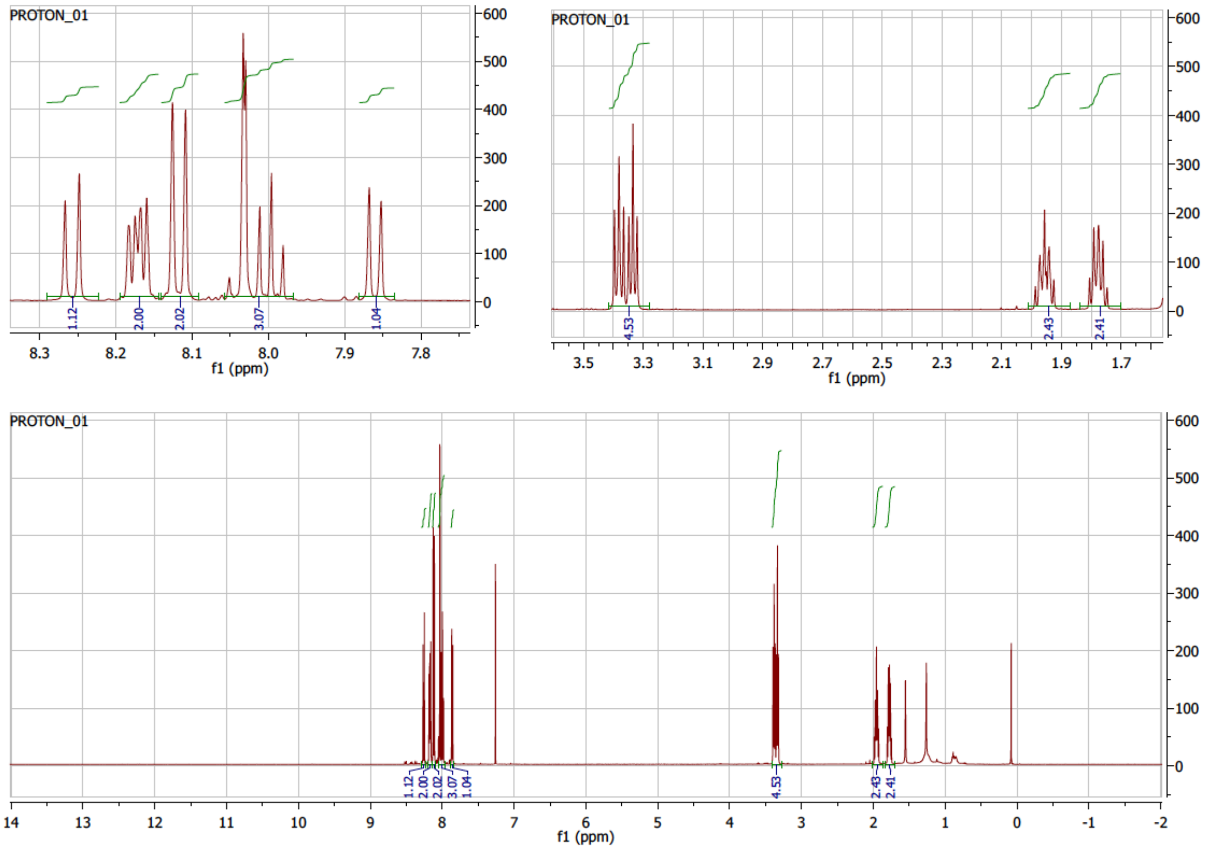

Figure S3. ¹H-NMR spectrum of 1-(4-azidobutyl)pyrene **3** in $CDCl_3$.

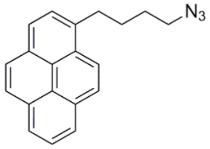

3

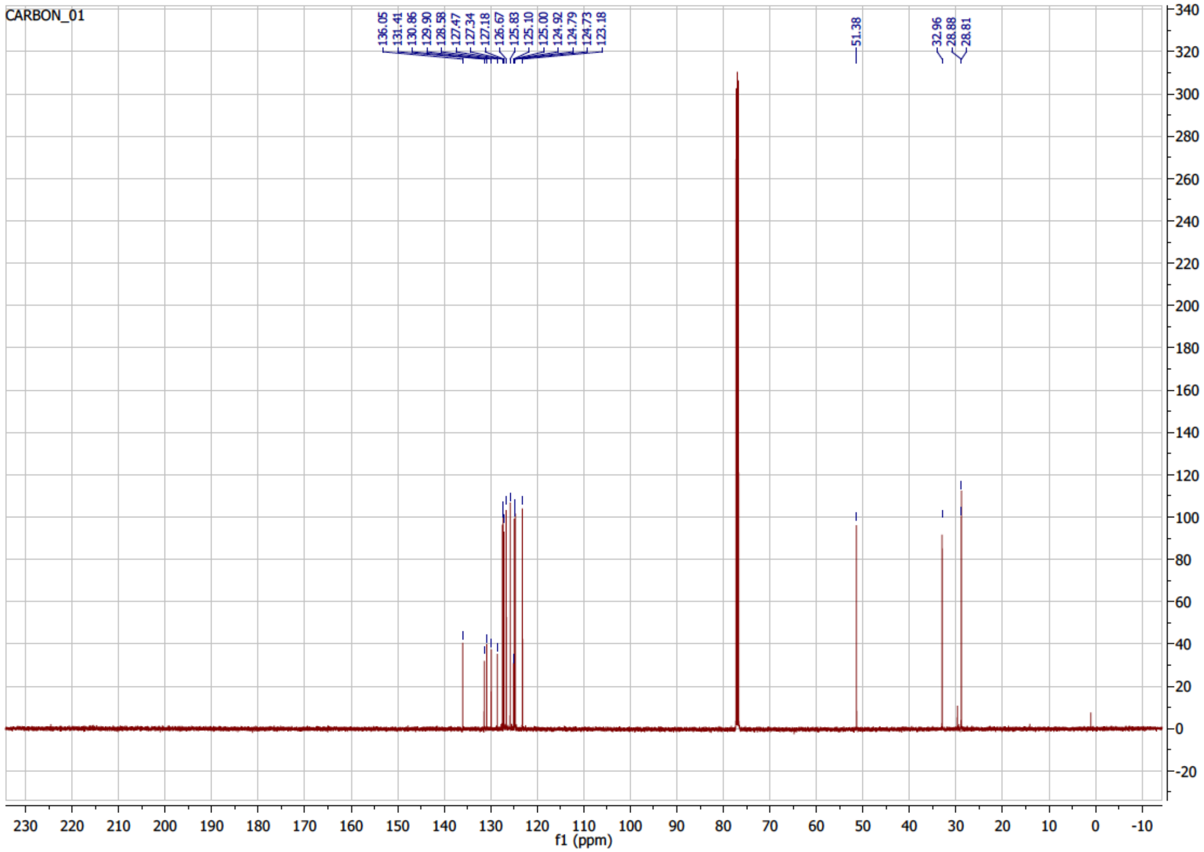

Figure S4. ^{13}C-NMR spectrum of 1-(4-azidobutyl)pyrene **3** in $CDCl_3$.

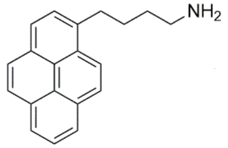

4

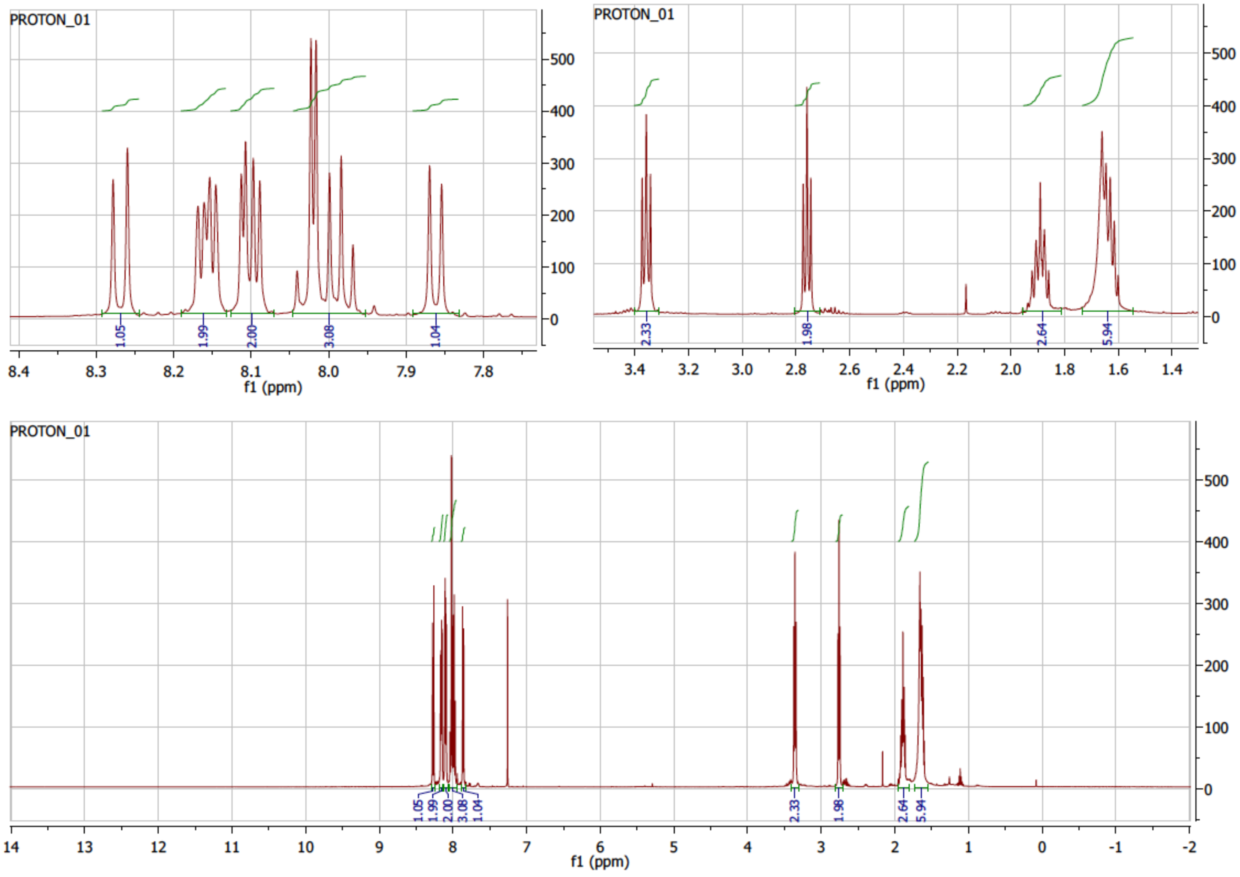

Figure S5. ^{1}H-NMR spectrum of 4-(1-pyrenyl)butanamine **4** in $CDCl_3$.

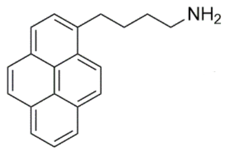

4

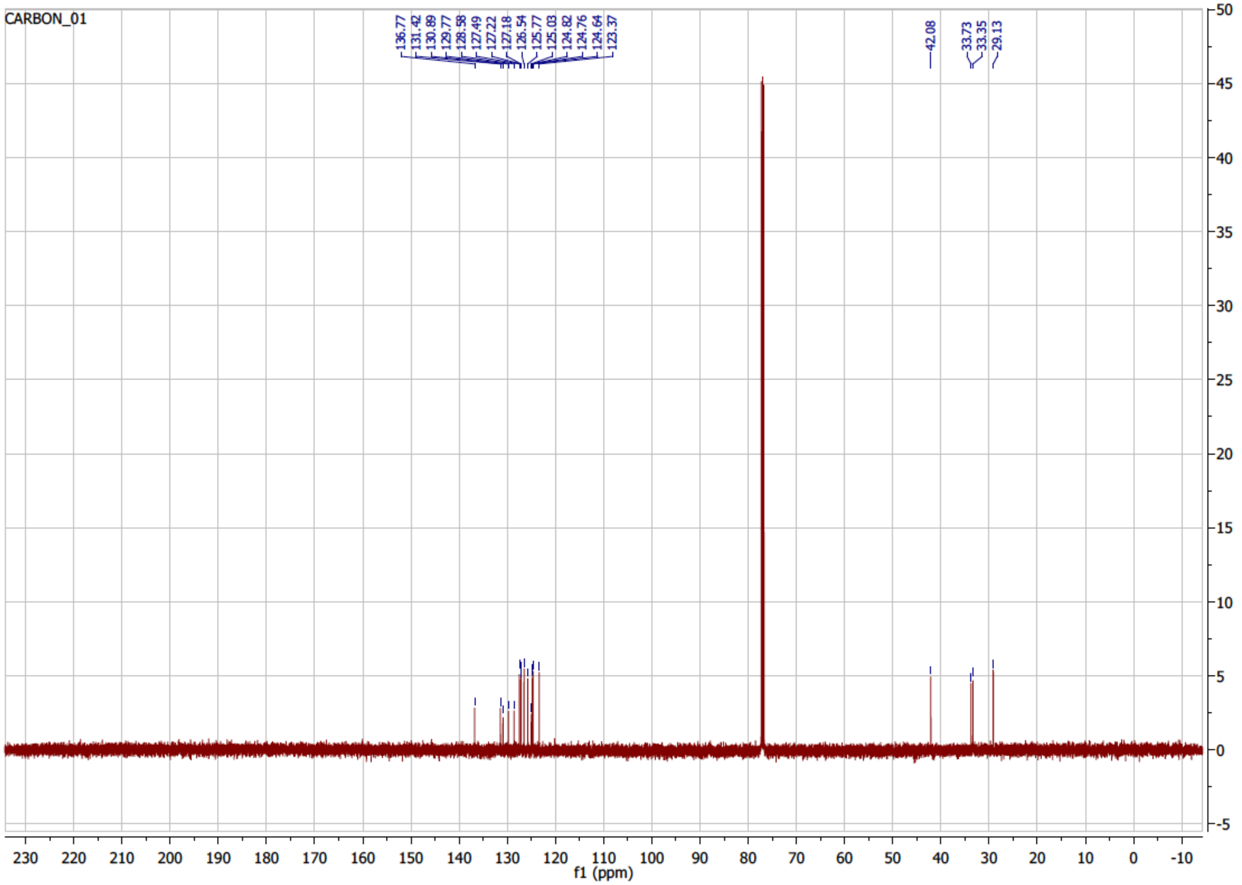

Figure S6. ^{13}C-NMR spectrum of 4-(1-pyrenyl)butanamine **4** in $CDCl_3$.

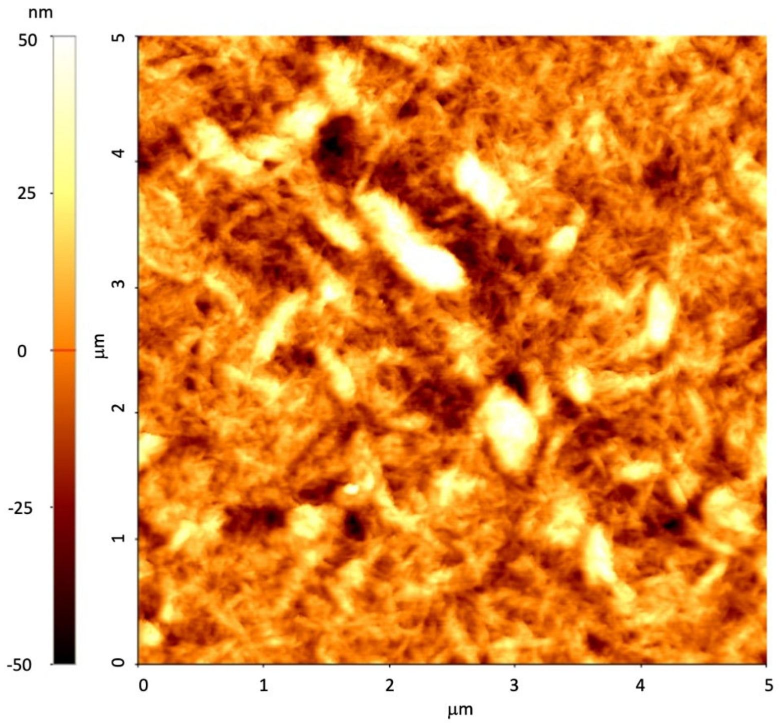

Figure S7. AFM 5x5 µm² topography recorded on a film of Pyrene modified S_CNC **7a** deposited on quartz from water.

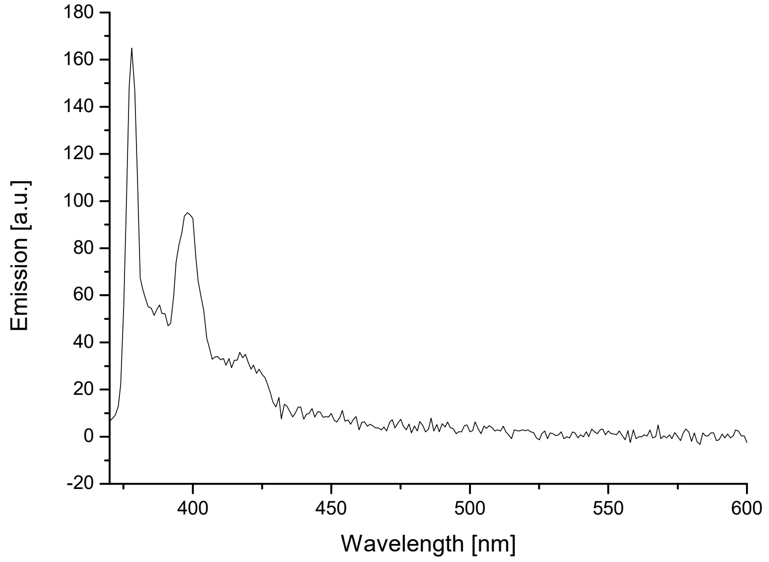

Figure S8. 4-(1-pyrenyl)butanamine **4** emission spectrum in water.

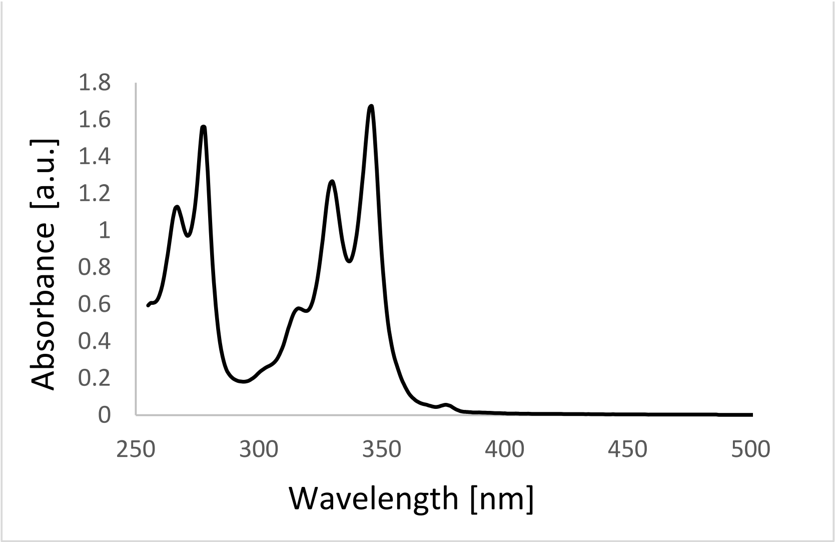

Figure S9. 4-(1-pyrenyl)butanamine **4** UV-vis absorption spectrum in DMSO.

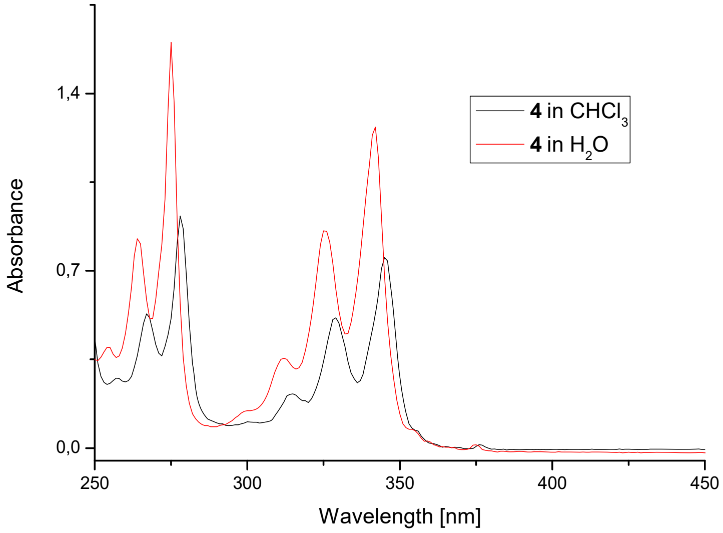

Figure S10. 4-(1-pyrenyl)butanamine **4** UV-vis absorption spectra in $CHCl_3$ and water.

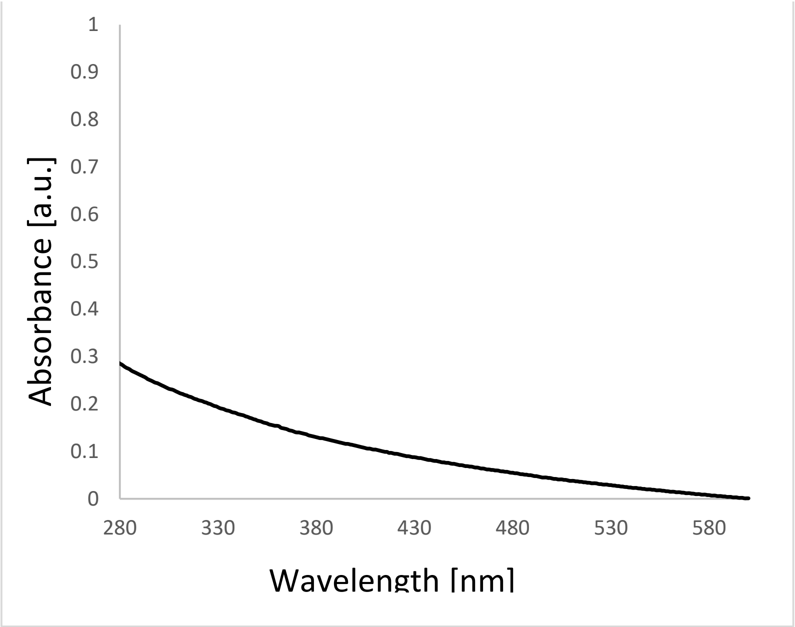

Figure S11. S_CNC UV-vis **5a** absorption spectrum in DMSO.

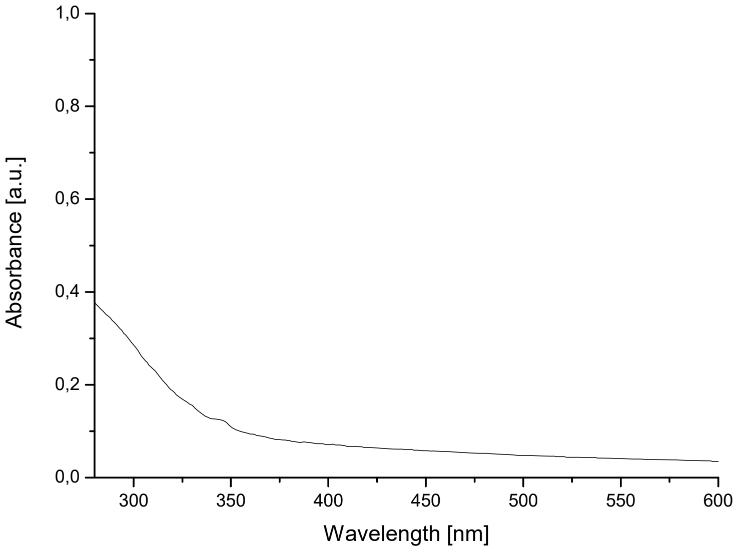

Figure S12. Pyrene modified S_CNC **7a** UV-vis absorption spectrum in DMSO.
